# Clinical Use of Hydrogen Sulfide to Protect Against Intimal Hyperplasia

**DOI:** 10.3389/fcvm.2022.876639

**Published:** 2022-04-11

**Authors:** Diane Macabrey, Alban Longchamp, Sébastien Déglise, Florent Allagnat

**Affiliations:** ^1^Department of Vascular Surgery, Lausanne University Hospital, Lausanne, Switzerland; ^2^Department of Biomedical Sciences, University of Lausanne, Lausanne, Switzerland

**Keywords:** restenosis, hydrogen sulfide (H_2_S), intimal and medial thickening, vascular SMCs, intimal hyperplasia

## Abstract

Arterial occlusive disease is the narrowing of the arteries *via* atherosclerotic plaque buildup. The major risk factors for arterial occlusive disease are age, high levels of cholesterol and triglycerides, diabetes, high blood pressure, and smoking. Arterial occlusive disease is the leading cause of death in Western countries. Patients who suffer from arterial occlusive disease develop peripheral arterial disease (PAD) when the narrowing affects limbs, stroke when the narrowing affects carotid arteries, and heart disease when the narrowing affects coronary arteries. When lifestyle interventions (exercise, diet…) fail, the only solution remains surgical endovascular and open revascularization. Unfortunately, these surgeries still suffer from high failure rates due to re-occlusive vascular wall adaptations, which is largely due to intimal hyperplasia (IH). IH develops in response to vessel injury, leading to inflammation, vascular smooth muscle cells dedifferentiation, migration, proliferation and secretion of extra-cellular matrix into the vessel’s innermost layer or intima. Re-occlusive IH lesions result in costly and complex recurrent end-organ ischemia, and often lead to loss of limb, brain function, or life. Despite decades of IH research, limited therapies are currently available. Hydrogen sulfide (H_2_S) is an endogenous gasotransmitter derived from cysteine metabolism. Although environmental exposure to exogenous high H_2_S is toxic, endogenous H_2_S has important vasorelaxant, cytoprotective and anti-inflammatory properties. Its vasculo-protective properties have attracted a remarkable amount of attention, especially its ability to inhibit IH. This review summarizes IH pathophysiology and treatment, and provides an overview of the potential clinical role of H_2_S to prevent IH and restenosis.

## Introduction

Prevalence of arterial occlusive disease continues to rise worldwide, largely due to the combination of aging, smoking, hypertension, and mostly diabetes mellitus (1–3).

Vascular surgery, open or endovascular, remains the only treatment for advanced arterial occlusive disease. However, the vascular trauma associated with the intervention eventually lead to secondary occlusion of the injured vessel, usually referred to as restenosis.

The overall incidence of restenosis varies greatly depending on the initial clinical presentation and the anatomic pattern of disease (e.g., coronary vs. femoro-popliteal vs. infra popliteal etc.). Overall, for open surgeries such as bypass and endarterectomy, the rate of restenosis after 1 year ranges between 20 and 30% (4). For endovascular approaches, the rate of restenosis following plain old balloon angioplasty (POBA) ranges from 30 to 60%, depending on location (5). In coronary arteries, the use of bare metal stents (BMS) lowered the rate of restenosis to 17–41% (5). In stented peripheral arteries, restenosis occurs in up to 51% of the patients 1 year after the surgery (6).

The most recent advances in the treatment of restenosis rely on the use of drug-coated balloons (DCB) and drug-eluting stents (DES), which nowadays represent a first line therapy in many endovascular approaches to treat short lesions in coronary or femoral arteries. The most used drug is the anti-tumor chemotherapy Paclitaxel (Taxol™). Several paclitaxel-coated balloons and eluting stents with various formulations and different dose of paclitaxel demonstrated superiority to POBA (7–9) or BMS (7, 10). Overall, the arrival of DES and DCB reduced the incidence of restenosis below 10% in coronary arteries (11), although restenosis has been delayed rather than suppressed (12). DES also require prolonged antiplatelet therapy and hinder future surgical revascularization. In peripheral *below the knee* small arteries, the use of DCB is controversial, and stents are not recommended due to the risk of thrombosis (13). In December 2018, Katsanos and colleagues reported, in a systematic review and meta-analysis, an increased risk of all-cause mortality following application of paclitaxel−coated balloons and stents in the femoropopliteal artery (14). Other groups recently confirmed these findings using the same data (15, 16). However, other meta-analyses did not find any association between paclitaxel devices and long-term survival, despite similar target populations and vessel segments (17–21). These reports questioned the widespread use of paclitaxel for the treatment of restenosis (22), and supports the need to develop other approaches or use other molecules. In coronary intervention Sirolimus is increasingly used (23), and new devices are under evaluation to validate the use of sirolimus-coated devices in *below the knee* peripheral arteries (24). Recent studies even report the safety and efficacy of biodegradable polymer sirolimus-eluting stent (25, 26).

**FIGURE 1:**
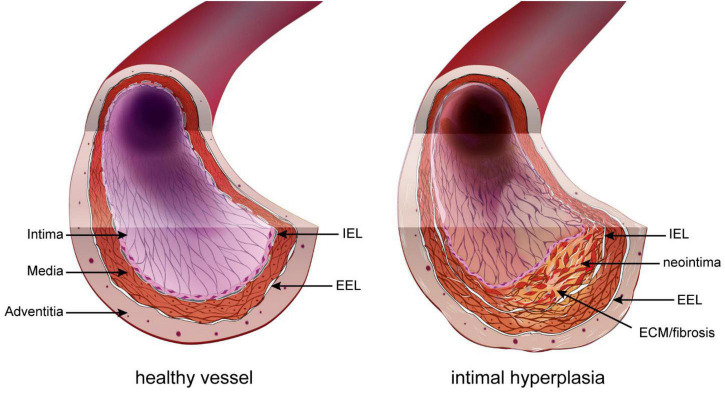
Intimal hyperplasia. In the healthy vessel (left), the inner intimal layer is composed only of the endothelium (pink layer). Under the endothelium sits the basement layer and internal elastic lamina (IEL). The media layer is composed of elastic fibers and smooth muscle cells (SMC). The outer part of the media and adventitia are separated by the exterior elastic lamina (EEL). Restenosis following vascular surgery is the formation of a neointima layer between the IEL and endothelium. It is composed of proliferating SMC-like cells of different origin and extracellular matrix (ECM).

Restenosis has various origins, such as secondary growth of atherosclerotic lesions or inward remodeling. However, it is due mostly to intimal hyperplasia (IH), a process whereby a “neointima” layer is formed between the internal elastic lamina (IEL) and the endothelium (Figure 1). IH is a known complication of all types of vascular procedures, including arterial bypass, angioplasty, stenting, and endarterectomy. Stenosis due to IH is also a major limitation of arteriovenous fistulas for hemodialysis patients, arteriovenous grafts, and other vascular accesses. The progressive growth of the neointima layer causes both an outward and an inward remodeling of the vessel wall, leading to a narrowing of the lumen, and eventually leads to impaired perfusion of downstream organs.

All current strategies to limit IH such as Paclitaxel and Sirolimus target cell proliferation. Paclitaxel is a chemotherapeutical agent that stabilizes microtubules, thereby preventing cell division (mitosis). High dose or prolonged exposure to paclitaxel may also lead to apoptotic cell death (27). Sirolimus inhibits the mammalian target of rapamycin (mTOR), a master regulator of cell growth and metabolism (28). However, targeting cell proliferation to reduce IH also impairs re-endothelization. Endothelium repair is crucial to limit inflammation, remodeling and IH. Poor endothelial repair also prolongs the need for anti-thrombotic therapies. Therefore, there is a need for new strategies to inhibit IH while promoting endothelium recovery. In that regard, the gasotransmitter hydrogen sulfide (H_2_S) possesses interesting properties.

H_2_S is a gasotransmitter derived from cysteine metabolism (29). Circulating H_2_S levels are reduced in humans suffering from vascular occlusive disease (30, 31) and pre-clinical studies using water-soluble sulfide salts such as Na_2_S and NaHS have shown that H_2_S has cardiovascular protective properties [reviewed in Zhang et al. (29)], including reduction of IH in various models (32–35).

In this review, we present the pathophysiology of IH and the clinical potential of H_2_S against IH. The pleiotropic benefits of H_2_S on the cardiovascular system are described, and the interesting possibilities to target the multifactorial process leading to IH using H_2_S are discussed.

## The Pathophysiology of Intimal Hyperplasia

The basic structure of large vessels (vein and artery) include three concentric layers: intima, media, and adventitia. The intima layer, also called endothelium, is the inner section of the vessel and is made of a single layer of endothelial cells (EC). The media is composed primarily of vascular smooth muscle cells (VSMC) and connective tissue made of collagen, elastin, and proteoglycans. The outermost adventitial layer is composed primarily of collagen and fibroblasts. In arteries, the intima and media layers are separated by a layer of elastic fibers called the internal elastic lamina (IEL), while the media and adventitia layers are separated by a second layer of elastic fibers called the external elastic lamina (EEL) (36). IH, also called neointima, develops between the intima and the IEL. The IH process is triggered in response to the injury to the blood vessel during surgery (37). This new layer is made of SMC-like cells and proteoglycan-rich extracellular matrix (ECM) (Figure 1).

### Endothelial Dysfunction or Lesion

**FIGURE 2:**
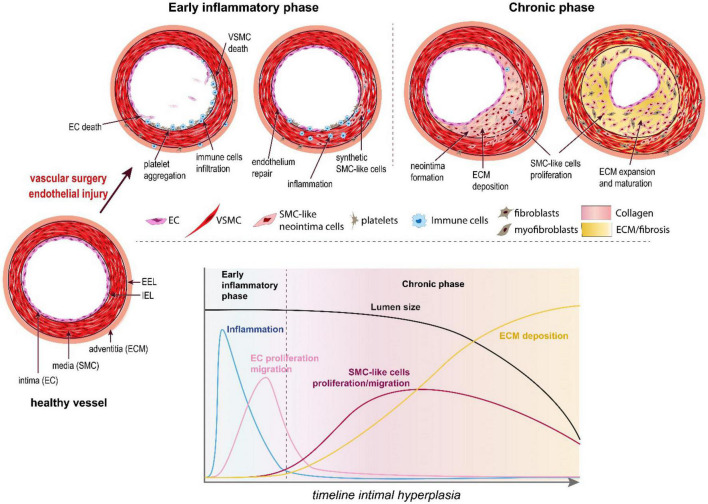
The pathophysiology of intimal hyperplasia. IH is triggered by endothelial injury, which activates platelets aggregation, and recruitment and activation of immune cells in the arterial wall (early inflammatory phase). The platelets and immune cells release cytokines, chemokines and growth factors, which stimulate a wound healing response mediated by SMC-like cells (mostly synthetic SMC derived from medial VSMC and myofibroblasts derived from adventitial fibroblasts). These synthetic SMC-like cells proliferate and migrate under the internal elastic lamina (IEL), forming the neointima layer. Long after inflammation is resolved and the endothelium is repaired (chronic phase), these cells continue to secrete extracellular matrix (ECM), leading to the progressive narrowing of the lumen.

Located at the contact between the blood and the vessel wall, the EC maintain a non-thrombogenic surface and regulate the vasomotor activity (vasodilation and vasoconstriction) of vessels. In arteries, EC require high laminar shear stress to maintain proper function, i.e., secrete anti-coagulation and vasodilation agents, mainly nitric oxide (NO) and prostacyclins (36). However, the hemodynamic forces are not uniform throughout the vascular system. In straight segments of arteries, blood flow is laminar and shear stress is high. However, at bifurcations, curvatures, or other regions with complex geometry, blood flow is disturbed and turbulent. These abnormal patterns of “low” shear stress induce “endothelial dysfunction” or “endothelium activation.” Because of these disturbed arterial flow patterns, nearly all humans develop benign IH, also referred to as diffuse intimal thickening, around vessel bifurcations or in curved sections of arteries. This type of lesion serves as a precursor for the development of atherosclerosis by facilitating local inflammatory reaction and entrapment of LDL in the vessel wall (38). Inevitably, these “weak” spots of the vascular system are the sites of primary occlusion by atherosclerotic plaques that require vascular interventions. Any vascular surgery destroys the endothelial layer, furthering endothelium damage on those existing weak spots. This is the case for balloon angioplasty, stenting, or endarterectomy, which directly target the site of the atherosclerotic plaques. In the case of bypass surgery and arteriovenous fistulas, the surgery damages the endothelium while creating new regions of disturbed arterial flow patterns, which will foster IH lesions (Figure 2).

Endothelial dysfunction or injury following surgery results in loss of eNOS, the enzyme producing nitric oxide (NO), a gasotransmitter maintaining healthy vessel function. Reduced NO production promotes vasoconstriction, platelet aggregation and recruitment/activation of resident and circulating inflammatory cells (mostly macrophages). The activated EC, recruited platelets and immune cells secrete cytokines and chemokines, which trigger a pro-inflammatory response (Figure 2). In addition, these cells secrete growth factors, including platelet-derived growth factor (PDGF), basic fibroblast growth factor (bFGF), transforming growth factor beta 1 (TGF-β) and thromboxane A2. Downstream of those multiple growth factors, cytokines and chemokines, the Mitogen-activated protein kinase (MAPK) pathway, including extracellular signal-regulated kinase (ERK), c-Jun-N-terminal kinase (JNK) and p38 mitogen-activated protein kinases, plays a major role in VSMC migration and proliferation in SMCs and fibroblasts. Other signals derived from oxidative stress also regulate the p38 MAPK and JNK pathways (39, 40).

Together, the secretion of these factors and the loss of NO promote vessel remodeling and reprogramming of cells composing the media and adventitia layers, leading to the formation of a neointima between the endothelium and the IEL (41) (Figures 1, 2).

### The Origin of Neointimal Cells

IH is mostly formed by proliferating VSMC originating from dedifferentiated contractile medial VSMC. Unlike other terminally differentiated cells of the myogenic lineage, such as cardiac and skeletal muscle cells, adult VSMC are highly plastic and capable of phenotypic alterations in response to their environment. Modulation of VSMC from a quiescent “contractile” phenotype to a proliferative “synthetic” phenotype is important for vascular injury repair, but is also a key factor in the pathogenesis of IH (42).

Upon vascular injury, the growth factors (PDGF-BB, bFGF), chemokines (SDF-1α, MCP-1) and cytokines (TNF-α, IL-1β) secreted by activated EC, platelets and immune cells stimulate ECM production and secretion, and reduce the expression of the contractile VSMC markers smooth muscle alpha-actin (α-SMA), smooth muscle myosin heavy chain (SM-MHC), calponin, and smooth muscle 22 alpha (SM22α) (42–44). In addition, the “activated” VSMC themselves produce cytokines such as TNFα and MCP-1, leading to positive feedback cascades of enhanced VSMC migration and proliferation (42). The main signaling pathway consistently shown to play a major role in SMC-like cells reprogramming and proliferation/migration is the MAPK pathway, mainly ERK1,2, JNK, and p38 (39, 40).

However, it is now accepted that neointimal VSMCs are phenotypically heterogeneous and that their origin and identity is diverse (45). After medial VSMC, the most abundant cell involved in neointima formation are probably myofibroblasts. Myofibroblasts originate from quiescent fibroblasts in the adventitia, which have converted into proliferating cells expressing several SMC markers such as α-SMA, SM-22α and calponin (46, 47) (Figure 2). Studies also support the existence of populations of mesenchymal stem cells or multipotent progenitor cells within the vessel wall, especially the adventitia layer, which could give rise to the fibroblasts and SMC-like cells found in the neointima layer (48, 49). Further studies identified similar progenitor cells in human arterial and venous tissue (50–52), suggesting a role for these cells in arterial remodeling and IH.

Neointimal cells may also arise from circulating progenitor cells or from the bone marrow (48, 53). However, the contribution of circulating progenitor cells to IH seems to depend upon the model and the type of injury (54). Similarly, studies in the atherosclerosis field yield conflicting results as to whether neointimal SMC-like cells originate from the bone marrow [reviewed in details in Albiero et al. (55)]. Of note, in human, it has been impossible to evaluate the role of circulating progenitors and *ex vivo* studies using human vessels clearly demonstrated that IH forms in a vessel self-sufficient manner, independently of circulating factors (56, 57). Drawing conclusion from these studies remains challenging given the small number of studies and the variety of experimental models and methodology, especially the methods and markers employed to isolate and identify “progenitor cells.” Recent studies of VSMC lineage in the context of atherosclerosis suggest that up to 80% of SMC-derived cells in the plaques do not express the classic SMC markers α-SMA, but express macrophage markers (CD68, LGALS3), mesenchymal stem cell marker (Sca 1) or myofibroblats markers (PDGFR-β). These cells all express KLF4, a major stem cell and differentiation mediator [reviewed in Allahverdian et al. (45)]. These new evidences underscore how little is known about the identity and origin of the cells responsible for the formation of IH. Advanced techniques of single cell lineage may shed new lights on key questions in the field. Clearly, further work is required to better characterize the cells composing the neointima layer in patients.

Once the neointima starts to form, the arrival and proliferation of SMC-like cells secreting important amount of ECM progressively expand the neointima layer. At first, this expansion is compensated by an outward remodeling of the vessel under the pressure of the blood flow to maintain the lumen area. The vessel gradually thickens as fibrosis sets in. Eventually, the resistance of the arterial wall exceeds the parietal pressure and the neointima extends inward, leading to a narrowing of the lumen and impaired blood flow (Figure 2).

## Hydrogen Sulfide

### Endogenous Hydrogen Sulfide Production

The discovery of an endogenous pathway releasing H_2_S in mammalian tissues came long after the discovery of NO, in 1960 with the work of Du Vigneaud, who investigated the oxidation of sulfur-containing amino acids in tissues. He discovered a new metabolic pathway involving the inter-conversion of cysteine and homocysteine and termed this pathway “transsulfuration.” Specifically, H_2_S is produced by two pyridoxal 5’-phosphate (PLP) dependent enzymes, cystathionine γ−lyase (CSE) and cystathionine β−synthase (CBS). Two additional PLP-independent enzymes, 3-mercaptopyruvate sulfurtransferase (3-MST) and cysteine aminotransferase (CAT) generate sulfane sulfur that can be further processed into H_2_S. 3-MST and CAT are expressed ubiquitously, whereas CBS and CSE display more tissue-specific patterns of expression. Thus, CBS is the only PLP-dependent enzyme expressed in the brain, while CSE is more prominent in the cardiovascular system. In the kidney and liver, both CSE and CBS are highly expressed. Although the enzymes and pathways responsible for endogenous H_2_S production are well defined, little is known about their regulation and their relative contributions to H_2_S and sulfane sulfur levels (e.g., polysulfides, persulfides, thiosulfate) in the circulation and in tissues under normal and disease conditions.

All H_2_S-synthesizing enzymes have been reported to be expressed by cardiovascular cells. The study of CSE^–/–^ mice demonstrated impaired endothelium-dependent vasorelaxation, with no apparent dysfunction at the level of VSMC (58). Furthermore, CSE seems sufficient to observe H_2_S-mediated vasodilation (59, 60). These observations strongly promoted the idea that CSE is the main H_2_S-producing enzyme in the cardiovascular system at the level of EC. However, other reports suggest a key role of 3-MST, along with CAT, in H_2_S production in the vascular endothelium (61). In contradiction to this early report, studies performed using CSE^–/–^ mice generated on a pure C57BL/6J genetic background by the group of Prof. Isao Ishii failed to show impaired endothelial function and hypertension (62, 63). In addition, most studies of endogenous H_2_S inhibition rely of the use of high concentrations of propargylglycin (PAG) to inhibit CSE. At these concentrations, PAG may also inhibit CBS, as well as other non-specific targets. Bibli et al. recently demonstrated that CSE expression is negatively regulated by shear stress, as opposed to eNOS in the mouse aorta (64, 65). This is in line with a previous study showing that only disturbed flow regions show discernable CSE protein expression after carotid artery ligation in the mouse (66).

### Cellular Effects of Hydrogen Sulfide

H_2_S contributes to the homeostasis of numerous systems, including the cardiovascular, neuronal, gastrointestinal, respiratory, renal, liver and reproductive systems (67).

The chemical nature of the molecules responsible for the biological activity of H_2_S remains elusive. HS^–^, polysulfides and sulfates have all been shown to affect a variety of signaling pathways and biological responses. The sulfur atom is a very potent electron acceptor/donor and H_2_S can undergo complex oxidation, yielding thiosulfate, sulfenic acids, persulfides, polysulfides and sulfate (68). These oxidative products are likely mediating the principal mechanism through which H_2_S exerts its biological actions: post-translational modification of proteins, known as persulfidation. Persulfidation is a chemical reaction whereby a persulfide group (RSSH) is formed on reactive cysteine residues of target proteins (68, 69). Since H_2_S has the same oxidation state as cysteine residues, a redox reaction cannot occur. Cysteine residues or H_2_S have to be oxidized first (for instance in the form of polysulfides H_2_S_n_). In 2009, Mustafa et al. performed LC/MS/MS analysis on liver lysates after NaHS treatment and identified 39 proteins that were persulfidated. Amongst them, they identified GAPDH, β-tubulin, and actin. Interestingly, these proteins were not persulfidated in the liver of mice lacking CSE (CSE KO) (70). Furthermore, new high throughput techniques allowing global assessment of post-translational modification of cysteinyl thiols (-SH) to persulfides (-SSH) demonstrated extensive cysteine residues persulfidation in response to various H_2_S donors across various experimental designs (71–73).

### Hydrogen Sulfide in Intimal Hyperplasia

Few studies directly assessed the effects of endogenous or exogenous H_2_S on IH. CSE expression and activity are reduced after balloon-injury in a rat model of IH (32). CSE expression and activity, as well as free circulating H_2_S, are also reduced in human suffering from vascular occlusive diseases (30, 74). We recently demonstrated that, in patient undergoing vascular surgery, circulating H_2_S levels were associated with long-term survival (75), suggesting low H_2_S production as a risk-factor for cardiovascular diseases. Mice lacking CSE show a significant increase in IH formation as compared to WT mice in a model of carotid artery ligation (34, 76). On the contrary, CSE overexpression decreases IH formation in a murine model of vein graft by carotid-interposition cuff technique (77). Similarly, NaHS administration limits the development of IH in *in vivo* models in rats (32), rabbits (33), and mice (34), and in human great saphenous vein segments *ex vivo* (35).

**FIGURE 3:**
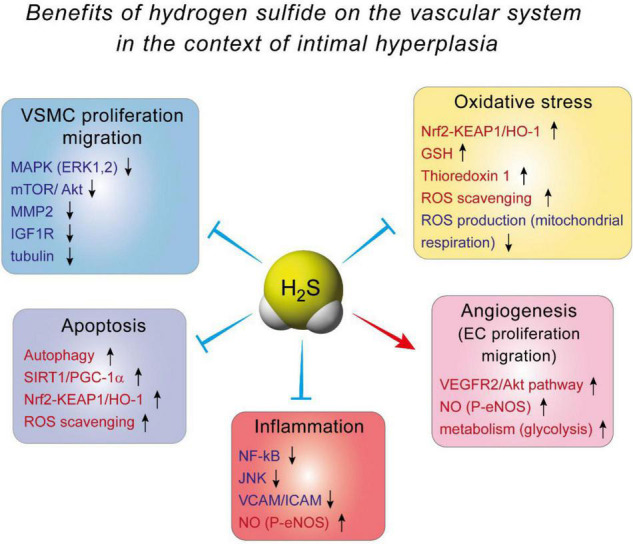
The benefits of hydrogen sulfide on the vascular system in the context of intimal hyperplasia.

The effect of H_2_S against IH is probably mediated by inhibition of VSMC proliferation and migration. Indeed, it was demonstrated, using BrdU and TUNEL assays, that H_2_S supplementation or CSE overexpression decreases VSMCs proliferation and increases VSMCs apoptosis, respectively (33, 35, 78). VSMCs isolated from Cse^–/–^ mice exhibit more motility than their WT counterpart, and blocking CSE activity using PAG in WT VSMCs increases cell migration (34, 79). The mechanisms whereby H_2_S affect VSMCs are not fully understood. In mouse VSMC, H_2_S has been shown to modulate the MAPK pathway, especially ERK1, 2 (32), and calcium-sensing receptors (80, 81). In addition, H_2_S may limit MMP2 expression and ECMs degradation, preventing migration of VSMCs from the media to the intima (34, 79). In human VSMC, we recently reported that the H_2_S donor Zofenopril decreases the activity of the MAPK and mTOR pathways, which correlates with reduced VSMC proliferation and migration (82). We also showed that the H_2_S donor salt NaHS, as well the thiol source sodium thiosulfate, inhibit microtubule polymerization, which results in cell cycle arrest and inhibition of proliferation and migration in primary human VSMC (76) (Figure 3).

Other studies also inform on potential mechanism of action of H_2_S on VSMC. The first evidence of H_2_S being a gasotransmitter comes from the consistent observation across species and vascular beds that H_2_S and other derived products induce vasodilation. H_2_S triggers vasodilation mostly *via* persulfidation of several ion channels such as K_ATP_, voltage and Ca^2+^-activated K^+^ channels (59, 60, 83). By reducing extracellular Ca^2+^ entry, H_2_S improves VSMC relaxation. Despite the obvious fact that improved vasorelaxation may be beneficial in the context of IH, these channels may also directly regulate cell proliferation in VSMC. For instance, the anti-diabetic and K_ATP_ channel blocker glibenclamide has been shown to reduce VSMC proliferation in a recent study (84).

### Other Benefits of Hydrogen Sulfide in the Cardiovascular System

#### Hydrogen Sulfide Stimulates Endothelial Cell Proliferation and Migration

Vascular surgery invariably leads to injury of the operated vessels. The single-cell endothelial barrier is very vulnerable and severely injured during surgery, which promotes inflammation and reprogramming of the adventitial and medial cells. The ability of endothelial cells to proliferate and migrate to restore the endothelial layer of the vessel is a key step in the resolution of post-operative inflammation to limit IH and restenosis. Unfortunately, EC are often neglected in strategies to reduce the formation of IH.

Although it has never been demonstrated in the context of IH, a large amount of studies established that H_2_S and polysulfides stimulate EC function and angiogenesis. Exogenous H_2_S treatment stimulates EC growth, motility and organization into vessel-like structure *in vitro*. On the contrary, inhibition of H_2_S biosynthesis, either *via* pharmacological inhibitors or *via* silencing of CSE, CBS or 3MST, reduces EC growth, migration and vessel-like structure formation (85, 86). Further *in vivo* studies of chicken chorioallantoic membranes (CAM) treated with the CSE inhibitor PAG suggest that CSE is important for vessel branching and elongation (87). Matrigel plug angiogenesis assay also confirmed the importance of CSE and H_2_S in vascular endothelial growth factor (VEGF)-induced angiogenesis (88, 89).

Several mechanisms have been proposed to explain H_2_S-induced angiogenesis. First and probably foremost, H_2_S stimulates the VEGF pathway in EC, through sulfhydration of the VEGF receptor VEGFR2, increasing its dimerization, autophosphorylation and activation (90). Interestingly, short term exposure of human EC to VEGF increases H_2_S production (87), suggesting a positive feedback loop of VEGF signaling through H_2_S. In addition, H_2_S promotes angiogenesis by inhibiting mitochondrial electron transport and oxidative phosphorylation, resulting in increased glucose uptake and glycolytic ATP production necessary to provide rapid energy for EC migration (91) (Figure 3).

H_2_S also promotes angiogenesis through its extensive interaction with the NO pathway. In endothelial cells, H_2_S may induce eNOS persulfidation at Cys433, which increases the phosphorylation of its activator site and stabilizes eNOS in its dimeric form (86, 92). H_2_S may also increase intracellular calcium levels, leading to increase eNOS activity and NO production (93, 94). Exogenous H_2_S donors have also been shown to stimulate the growth pathways Akt, p38 and ERK1/2, which all promote EC proliferation and migration (87, 89, 95). Finally, both H_2_S- and NO-induced angiogenesis require the other gasotransmitter (86, 92). Thus, the vascular effects of NO and H_2_S are interdependent and closely intertwined, with both gasotransmitter having direct and indirect effects on each other [for full review see Szabo et al. (96)]. Overall, the effect of H_2_S on EC may facilitate re-endothelization following vascular trauma, accelerating healing of the intima layer and limiting IH (Figure 3).

#### Hydrogen Sulfide Inhibits Inflammation

The surgical trauma and injury to the endothelial layer triggers inflammation, which contributes to IH. Many studies report anti-inflammatory properties of H_2_S, in particular in the context of atherosclerosis and cardiac failure [for full review see Pan et al. (97)]. Thus, H_2_S reduces adhesion and infiltration of pro-inflammatory cells and circulating levels of pro-inflammatory chemokines and cytokines in the ApoE^–/–^ mouse model of atherosclerosis (98, 99). Similarly, several reports document that H_2_S donors (NaHS, DATS, SG1002, STS) or CSE overexpression decrease leukocyte and neutrophil infiltration and cytokine production following ischemic injury in various models of myocardial infarction (100–105).

Mechanistically, evidence from EC and macrophages indicate that Nuclear factor kappa B (NFkB) inhibition seems to be the key to H_2_S anti-inflammatory effects (98, 106–108). NF-kB is a transcription factor and a master regulator of pro-inflammatory genes, including cytokines and cell adhesion molecules. NaHS inhibits NF-kB activity probably *via* persulfidation/stabilization of IkB (109), which controls NF-kB (p65) translocation to the nucleus. In EC, this leads to decreased expression of adhesion molecule VCAM and ICAM, thereby limiting recruitment of leukocyte to the aortic wall (98, 106, 108). NaHS also promotes a shift in macrophages to the M2, pro-resolution state (110). Moreover, NaHS and GYY increase eNOS phosphorylation, thereby improving NO production, which reduces inflammation (106, 111). Whether or not H_2_S-based strategies may reduce inflammation in the context of IH remains to be tested (Figure 3).

#### Hydrogen Sulfide Has Anti-oxidant Properties

Several studies in various models also consistently showed that H_2_S holds anti-oxidant properties. First, H_2_S can directly scavenge reactive oxygen species (ROS), such as superoxide anions O_2_^–^, at higher rates than other classic antioxidants such as GSH. However, since H_2_S physiologic concentration is in the nanomolar range whereas GSH is present in millimolar quantity, it is debatable whether H_2_S direct contribution to anti-oxidation is significant.

Actually, the effect of H_2_S probably arises from stimulation of anti-oxidant pathways, rather than *via* direct scavenging of ROS. First, H_2_S increases GSH production *via* modulation of the transsulfuration pathway. H_2_S interaction with GSH has been studied in details in the central nervous system, where GSH plays a major role in maintaining the homeostasis between anti-oxidant and ROS production [reviewed in details in Shefa et al. (112)]. In the vascular system, H_2_S persulfidates the glutathione peroxidase 1, which promotes glutathione synthesis and results in decreased lipid peroxidation in the aortic wall in the context of atherosclerosis (113). Second, numerous studies document that H_2_S promotes the Nrf2 pathway [reviewed in Corsello et al. (114)]. H_2_S promotes the Nrf2 anti-oxidant response *via* persulfidation of Kelch-like ECH-associated protein 1 (Keap1), which sequesters Nrf-2 in the cytosol. Keap1 persulfidation prompts dissociation from Nrf2, which induces the expression of several proteins, among which the major antioxidant protein heme oxygenase 1 (HO-1). This mechanism reduces oxidative stress, leading to reduced atherosclerosis in diabetic low density lipoprotein receptor (LDLR) knock –out mice (115), and cardioprotection in a model of ischemia-reperfusion injury (116, 117). Finally, H_2_S also stimulates thioredoxin 1 (Trx) expression, *via* silencing the expression of inhibitory protein Trx-interacting protein (TXNIP) (118, 119). Increased Trx is instrumental in the cardioprotective effects of H_2_S against ischemia-induced heart failure (118) (Figure 3).

Mitochondrial respiration is a major source of ROS (120, 121). H_2_S has a bell-shaped effect on mitochondrial respiration. At low nanomolar concentrations, sulfide quinone oxidoreductase (SQR) transfers electrons from H_2_S to the coenzyme Q in the Complex II of the electron transport chain, thereby promoting mitochondrial respiration. At higher concentrations, H_2_S binds the copper center of cytochrome c oxidase (complex IV), thereby inhibiting respiration and limiting ROS production (122).

These anti-oxidant properties of H_2_S may have a beneficial impact on IH, as ROS contribute to endothelial dysfunction and VSMC dedifferentiation (123, 124).

## Discussion: New Perspectives for the Treatment of Intimal Hperplasia Using Hydrogen Sulfide-Based Therapies

There is currently no clinically approved molecule exploiting the clinical potential of H_2_S. Most compounds available for research have poor translational potential due to their pharmacokinetic properties. Thus, the highly soluble unstable salts sodium hydrogen sulfide (NaHS) and disodium sulfide (Na_2_S) release H_2_S instantly in an uncontrollable manner, and thus have narrow clinical ranges. Other H_2_S-releasing molecules extracted from garlic such DATS (Diallyl trisulfide) and DADS (Diallyl disulfide), which have both been shown to possess vasoactive properties (125), are also very short lived and hard to stabilize (125). In the past years, several H_2_S-releasing compounds have been studied and developed for clinical purposes (Table 1).

**TABLE 1:** Clinical development of H_2_S-releasing compound.

Name	Description	Indications	Development phase	References
Zofenopril	ACE inhibitor combined to an H_2_S donor	Hypertension	Approved	(136, 137)
ATB-340	H_2_S-releasing derivative of low dose aspirin	Anti-thrombotic for chronic prevention of cardiovascular diseases and cancer chemoprevention	Dropped	(139)
ATB-352	H_2_S-releasing derivative of ketoprofen	NSAID	Pre-clinical	(153)
ATB-346 Otenaproxesul	H_2_S-releasing derivative of naproxen	NSAID; Gastric Ulcer, Osteoarthritis	Phase II NCT03978208 NCT03291418	(140)
S-Diclofenac	Derivative of Diclofenac combined to an H_2_S donor	NSAID	Pre-clinical; Dropped?	(141–143)
IK-1001	Injectable stable form of Na_2_S	Reduction of heart complications during coronary artery bypass graft	Stopped during phase 2 trial NCT00858936	(144)
SG1002	H_2_S-releasing prodrug	Heart failure	Phase 1 NCT02278276	(146)
Sodium thiosulfate	Inorganic sodium salt with thiosulfate ions	Calciphylaxis (ESRD) and cyanide poisoning	Approved NCT00568399 NCT01008631 NCT03150420 NCT02899364	(149, 154)

*NSAID, Non-steroidal anti-inflammatory drug; ESRD, End Stage Renal disease.*

### Hydrogen Sulfide-Based Therapies: Current Strategies

S-allylcysteine (SAC), an organosulfur compound present in abundance in garlic, has been shown to lower the mortality and reduce the infarct size in a rat model of acute myocardial infarction (126) and improves blood flow recovery after hindlimb ischemic injury in the mouse (127). S-propyl-L-cysteine and S-propargyl-L-cysteine, structural analogs of SAC found in garlic, have also been shown to slowly release H_2_S (128). Interestingly, S-propargyl-L-cysteine has been demonstrated to have both cardio protective (129, 130) and pro-angiogenic properties in preclinical models (131). However, despite the well-known cardiovasculo-protective properties of garlic, these active compounds are not currently used for clinical studies.

Another strategy used by pharmaceutical companies to harness the benefits of H_2_S has been to combine a H_2_S-releasing moiety with well-established parent compounds.

Zofenopril is one such product. Zofenopril is an ACE Inhibitors (ACEi) and a H_2_S donor combined (132). ACEi constitute one of the first-line class of antihypertensive drugs (133). Several clinical studies have shown that sulfhydrylated ACEi zofenopril has additional beneficial actions compared to non-sulfhydrylated ACEi such as enalapril or ramipril. Thus, zofenopril improves the clinical outcome of patients with different cardiovascular diseases such as acute myocardial infarction and congestive heart failure (134–137). We recently demonstrated that Zofenopril is more potent than Enalapril in reducing IH in a genetic model of hypertensive mice. In addition, it suppresses IH in normotensive condition, where other non-sulfhydrylated ACEi (Enalapril, Lisinopril and Quinapril) have no effect. Furthermore, Zofenopril prevents IH in an *ex vivo* model of IH in human saphenous vein. The effect of Zofenopril on IH correlates with reduced VSMC proliferation and migration and decreased activity of the MAPK and mTOR pathways (138).

*Antibe Clinicals*, a startup created around H_2_S-releasing compounds, synthesizes several H_2_S-releasing derivatives conjugated to NSAID for the treatments of pain and inflammation.^1^ ATB-340 is a H_2_S-releasing derivative of low-dose aspirin without the serious risk of gastrointestinal bleeding. Pre-clinical studies have demonstrated that ATB-340 caused negligible GI damage compared to low-dose aspirin (139). ATB 346, which is derived from the NSAID naproxen, was recently shown in a Phase 2B study to reduce GI toxicity compared to naproxen alone, with equivalent suppression of COX activity (140). The H_2_S-releasing diclofenac *S-Diclofenac* (ATB-337 or ACS-15), where H_2_S is linked to diclofenac *via* an ester bond, may also present advantages compared to classical Diclofenac (141, 142). *S-Diclofenac* has been shown to inhibit smooth muscle cell proliferation, and may play a role in restenosis in vascular injury (143). However, there was no further development of this compound.

The compound IK-1001, from the company Mallinckrodt, is an injectable stable form of Na_2_S (144). Despite a first phase I safety trial showing no adverse events, the development of IK-1001 was stopped by the company during a phase II efficacy trial in patients undergoing surgery for a coronary artery bypass graft (ClinicalTrials.gov ID: NCT00858936).

SG1002, initially developed by Kondo et al. (145), and further developed by the startup company Sulfagenix, is a prodrug releasing H_2_S. It has been tested on humans in the setting of congestive heart failure during one of the first phase 1 trial using a sulfide-based therapy to treat cardiovascular diseases. The results of this study were promising as SG1002 was able to restore sulfide and NO levels in patients with heart failure (146). However, additional studies are obviously required.

Sodium thiosulfate (STS; Na_2_S_2_O_3_) is an inorganic sodium salt containing thiosulfate ions in a 2:1 ratio. Pharmaceutical-grade STS is available and has been suggested to release H_2_S through non-enzymatic and enzymatic mechanisms (147, 148). STS is the treatment of choice for cyanide poisoning as thiosulfate is used by Rhodanese to convert cyanide to less toxic thiocyanate. Intravenous STS is also used to increase the solubility of calcium for the treatment of acute calciphylaxis, a rare vascular complication of patients with end-stage renal disease (149). Sodium thiosulfate is also under test in a number of clinical trials for the treatment of ectopic calcification (NCT03639779; NCT04251832; NCT02538939); to reduce ototoxicity in patients receiving cisplatin chemotherapy for standard risk hepatoblastoma (NCT05129748); in combination with chemotherapy to prevent low platelet count in patients with malignant brain tumors (NCT00075387). We also recently demonstrated that STS limits IH development *in vivo* in a model of arterial restenosis and in our *ex vivo* model of IH in human veins. STS treatment increases H_2_S bioavailability, which inhibits cell apoptosis and fibrosis, as well as VSMC proliferation and migration *via* microtubules depolymerization (76). Interestingly, an ongoing clinical study aims to evaluate the efficacy and safety of STS compared to placebo on myocardial infarct size in ST-segment elevation myocardial infarction (STEMI) patients treated with percutaneous coronary intervention (PCI) (NCT02899364).

The focal nature of IH lesions provide a window of opportunities for the use of local drug delivery using vascular medical devices. A number of approaches have been tested to apply treatment locally, including DCB and DES, as well as periadventitial drug delivery and targeted systemic therapies (150). Unlike current non-specific cytostatic drugs, local H_2_S delivery might provide a unique clinical opportunity to inhibit VSMCs proliferation while promoting ECs proliferation and endothelial repair. We recently developed and evaluated the clinical potential of an H_2_S-releasing biodegradable hydrogel to limit the development of IH in human veins. The thiol-triggered, controlled H_2_S release from peptide hydrogels provided sustained H_2_S concentrations over the period of hours, which inhibited VSMC proliferation and IH in human vein models more effectively than the sulfide salts (NaHS). The H_2_S-releasing peptide hydrogel also facilitated HUVEC proliferation and transmigration *in vitro*, which may promote re-endothelization, thereby supporting vascular repair (35). Recently, it was shown that a locally applicable gel containing the hydrogen sulfide releasing prodrug (GYY4137) mitigates graft failure and improve arterial remodeling in a model of vein graft surgery in the mouse (151).

### Hydrogen Sulfide-Based Therapies: Advantages and Limitations

IH is a complex process, involving multiple cell types and developing over the course of several years. IH is triggered by an acute endothelial dysfunction and associated pro-inflammatory response, which triggers a cascade of event leading to the formation of the neointima layer. The neointima slowly grows over the course of months to years, long after the acute inflammation is resolved and the endothelium repaired (Figure 2). H_2_S is unique in the context of IH because it can have beneficial impact on both the acute pro-inflammatory response and the chronic neointima growth. Thus, on the one hand, H_2_S limits inflammation and oxidative damages, while promoting EC proliferation and endothelium repair. On the other hand, H_2_S limits the proliferation and migration of synthetic SMC-like cells forming the neointima layer. In contrast, current strategies to reduce IH aggressively target cell proliferation, which also affect re-endothelization, prolonging inflammation and the need for anti-thrombotic therapies. Moreover, recent reports suggest that paclitaxel-releasing balloons and stents may have deleterious long-term effects, which is not surprising given that it is a cytotoxic chemotherapeutic agent.

Numerous drugs have been tested over the years to limit IH, demonstrating outstanding potential in pre-clinical studies in the small animal. Yet, in most trials, the pharmacologic treatment of restenosis failed to have a positive impact (150, 152). It is probable that the lack of efficacy in humans is, at least partly, due to insufficient drug delivery at the site of injury, as much higher dosages of drugs were generally used in animal models. It will be interesting to see whether H_2_S-based solutions can bridge the gap between benchtop and bedside. The first challenge will be to develop stable H_2_S-donor molecules allowing slow and sustained H_2_S release over the course of months/years. Such molecules are yet to be developed and will be hard to design given the instability and short half-life of H_2_S. Another challenge for either systemic or local release of H_2_S reside in the delivery system. The development of DCB and DES releasing paclitaxel or sirolimus led to innovative delivery systems. Gels, nanoparticles, multiple-layer coatings and biodegradable scaffolds have been invented to allow sustained drug release. It will be interesting to apply this knowledge to H_2_S-donor molecules. Eventually, the development of H_2_S-releasing balloons and stents could provide much-needed device to limit VSMC proliferation while promoting EC recovery. However, combining local delivery and systemic oral drug administration will probably be necessary to prevent IH successfully.

## Conclusion

Restenosis due to IH is recurrent and there is no efficient therapy. The neointima layer has a muscular and fibrotic rigid structure, which is hard to treat. Additional interventions to re-open the vessel invariably results in trauma, leading to further IH. DCB and DES improved the primary patency of vessels following endovascular surgeries but in-stent restenosis poses new challenges. Current strategies target cell proliferation to reduce IH, which also affect re-endothelization, prolonging the need for anti-thrombotic agents. Moreover, recent reports suggests that paclitaxel-releasing balloons and stents may have deleterious long-term effects.

These limitations warrant further research to better understand the molecular mechanisms of IH and develop new molecules limiting VSMC proliferation while stimulating EC proliferation and re-endothelization. Although H_2_S research is still in its infancy, ample evidence point to a protective role for this gaseous transmitter in the development of cardiovascular diseases. However, further animal studies are required to test the potential and safety of new H_2_S-based therapies. Understanding these questions will provide insightful knowledge about the biology of H_2_S and help design successful H_2_S-based therapies in the future.

## Author Contributions

FA and SD made the backbone. DM, FA, AL, and SD wrote and revised the manuscript. FA made the figures. All authors contributed to the article and approved the submitted version.

## Conflict of Interest

The authors declare that the research was conducted in the absence of any commercial or financial relationships that could be construed as a potential conflict of interest.

## Publisher’s Note

All claims expressed in this article are solely those of the authors and do not necessarily represent those of their affiliated organizations, or those of the publisher, the editors and the reviewers. Any product that may be evaluated in this article, or claim that may be made by its manufacturer, is not guaranteed or endorsed by the publisher.

## Footnotes

1
http://www.antibethera.com

## Abbreviations

3-MST: 3-mercaptopyruvate sulfurtransferase
AKT: Serine/Threonine Kinase 1
AOAA: aminooxyacetic acid
bFGF: Basic Fibroblast Growth Factor
CAT: cysteine aminotransferase
CBS: cystathionine β synthase
COX: cytochrome c oxidase
CSE: cystathionine γ lyase
DATS: Diacyltrisulfite
DES: Drug-Eluting Stents
DCB: Drug-Coated Balloons
EC: Endothelial Cells
ECM: Extracellular Matrix
EEL: external elastic lamina
Enos: Endothelial Nitric Oxide Synthase
ER: endoplasmic reticulum
ERK: Extracellular Signal-Regulated Kinase
ETHE1: ethylmalonic encephalopathy 1 protein
GSH: Reduced Glutathione
GSSG: oxidized GSH
ICAM1: Intercellular Adhesion Molecule 1
IEL: internal elastic lamina
IH: Intimal Hyperplasia
IL-1 β: Interleukin 1 beta
JNK: C-Jun N Terminal Kinase
KATP: ATP-sensitive K^+^
Keap1: kelch-like ECH-associated protein 1
LDH: lactate dehydrogenase
LDL: Low-Density Lipoprotein
MAPK: Mitogen-Activated Protein Kinase
MCP-1 (CCL-2): Monocyte Chemoattractant Protein-1
MMP: Matrix Metalloproteinase
NaHS: Sodium Hydrogen Sulfur
NF- K b: Nuclear Factor Kappa b
NO: Nitric Oxide
Nrf2: nuclear factor (erythroid-derived 2)–like 2
PCI: percutaneous coronary intervention
PCNA: Proliferating Cell Nuclear Antigen
PDGF: Platelet-Derived Growth Factor
Pdgfr- B: Beta-Type Platelet-Derived Growth Factor Receptor
PLP: pyridoxal 5’-phosphate
POBA: plain old balloon angioplasty
ROS: Reactive Oxygen Specie
SDF-1 α: Stromal Cell-Derived Factor 1
SM22 α (Tagln): Smooth Muscle 22 Alpha/Transgelin
SMA (ACTA2): Smooth Muscle Actin Alpha
SQR: sulfide quinone oxidoreductase
STEMI: ST-segment elevation myocardial infarction
STS: Sodium Thiosulfate
TNF- α: Tumor Necrosis Factor Alpha
TIMP: Tissue Inhibitors Of Metalloproteinase
Trx: thioredoxin
TXNIP: Trx-interacting protein
VCAM1: Vascular Cell Adhesion Protein 1
VSMC: Vascular Smooth Muscle Cells
WT: wild type.
